# Ferroptosis-related biomarkers for Alzheimer’s disease: Identification by bioinformatic analysis in hippocampus

**DOI:** 10.3389/fncel.2022.1023947

**Published:** 2022-11-16

**Authors:** Binyang Wang, Chenyang Fu, Yuanyuan Wei, Bonan Xu, Rongxing Yang, Chuanxiong Li, Meihua Qiu, Yong Yin, Dongdong Qin

**Affiliations:** ^1^Department of Rehabilitation Medicine, The Affiliated Hospital of Yunnan University, Kunming, China; ^2^School of Basic Medical Sciences, Yunnan University of Chinese Medicine, Kunming, China

**Keywords:** ferroptosis, Alzheimer’s disease, biomarkers, hippocampus, ASNS, SESN2

## Abstract

**Background:**

Globally, Alzheimer’s Disease (AD) accounts for the majority of dementia, making it a public health concern. AD treatment is limited due to the limited understanding of its pathogenesis. Recently, more and more evidence shows that ferroptosis lead to cell death in the brain, especially in the regions of the brain related to dementia.

**Materials and methods:**

Three microarray datasets (GSE5281, GSE9770, GSE28146) related to AD were downloaded from Gene Expression Omnibus (GEO) datasets. Ferroptosis-related genes were extracted from FerrDb database. Data sets were separated into two groups. GSE5281 and GSE9770 were used to identify ferroptosis-related genes, and GSE28146 was used to verify results. During these processes, protein–protein interaction (PPI), the Gene Ontology (GO), and Kyoto Encyclopedia of Genes and Genomes (KEGG) pathway enrichment analyses were conducted. Finally, the differentiated values of ferroptosis-related genes were determined by receiver operator characteristic (ROC) monofactor analysis to judge their potential quality as biomarkers.

**Results:**

Twenty-four ferroptosis-related genes were obtained. Using STRING (https://cn.string-db.org/) and Cytoscape with CytoHubba, the top 10 genes (*RB1, AGPAT3, SESN2, KLHL24, ALOX15B, CA9, GDF15, DPP4, PRDX1, UBC, FTH1, ASNS, GOT1, PGD, ATG16L1, SLC3A2, DDIT3, RPL8, VDAC2, GLS2, MTOR, HSF1, AKR1C3, NCF2*) were identified as target genes. GO analysis revealed that response to carboxylic acid catabolic process, organic acid catabolic process, alpha-amino acid biosynthetic process and cellular amino acid biosynthetic process were the most highly enriched terms. KEGG analysis showed that these overlapped genes were enriched in p53 signaling pathways, longevity regulating pathway, mTOR signaling pathway, type 2 diabetes mellitus and ferroptosis. Box plots and violine plots were created and verified to confirm the significance of identified target genes. Moreover, ROC monofactor analysis was performed to determine the diagnostic value of identified genes. Two genes (*ASNS*, *SESN2*) were subsequently obtained. For the tow genes, STRING was used to obtain the five related genes and determined enriched GO terms and KEGG pathways for those genes.

**Conclusion:**

Our results suggest that ASNS and SENS2 may serve as potential diagnostic biomarkers for AD and provide additional evidence regarding the essential role of ferroptosis in AD.

## Introduction

The number of people suffering from Alzheimer’s disease (AD) are increasing globally, and AD influences many aspects from daily lifestyle to family expenditures (Jia et al., 2020; Scheltens et al., 2021). AD is the most common cause of dementia, which may account for approximately 60–80% of cases (Cunningham et al., 2015; Scheltens et al., 2021; Zhang et al., 2021). Clinical characteristics of AD include neurodegeneration with symptoms of cognitive and functional impairment such as decreased memory and ability to perform activities of daily living, and decreased behavioral capacity. Thus, it is important and emergent to figure out how to diagnose and detect AD earlier (Arvanitakis et al., 2019; Scheltens et al., 2021). At present, the pathogenesis of AD focuses mainly on contributions from amyloid β (Aβ) and Tau. AD may be due to the production, aggregation, and accumulation of Aβ leading to neurotoxicity or to changes in normal Tau, or it may be due to Aβ inducing the pathological transmission of Tau (Shaw et al., 2007; Scheltens et al., 2021). Currently, dementia is diagnosed according to associated behavioral traits rather than biological characters, such as brain imaging, due to its complexity and various potential causes, including cerebral cardiovascular diseases, Lewy body disease and frontotemporal dementia (McKhann et al., 2011; Arvanitakis et al., 2019). Therefore, there is a current need to determine specific biomarkers that can be used for the clinical diagnosis of AD, and these biomarkers can also be used for the exploration of future disease mechanisms and to obtain the best therapeutic targets.

Scientists have deemed that the hippocampal brain region is responsible for the function of memory, learning and spatial information in animal and human research (Jarrard, 1995; Knierim, 2015). Exploration between the hippocampal region and AD and dementia is continuing. A research report in 1999 showed that the declination of memory with aging was related to changes in the hippocampal region in AD patients (Small et al., 1999). A review concluded that AD patients suffer impairment in emotional event memory, as viewed in the amygdalohippocampal interconnection, with the amygdala showing damage characteristic of atrophy, plaques and tangles (McDonald and Mott, 2017). Recently, researchers have found new insights into the relationships between AD and the hippocampal region, specifically advancing cell death and neurodegeneration.

In Dixon et al., 2012 officially proposed the conception of ferroptosis (an iron-dependent, oxidative, non-apoptotic cell death) for the first time, which started the formal study of the mechanism between ferroptosis and various diseases. Compared with research on the relationship between ferroptosis and cancer or tumors, the link between ferroptosis and neurological diseases, especially AD, has been poorly studied. Searching ferroptosis and AD in Pubmed revealed that the exploration of ferroptosis and AD began to focus in 2017, and the number of publications in the area has surged in recent years. In 2019, researchers surprisingly found that mice with high dietary iron exhibit neuronal loss by apoptosis, autophagy and ferroptosis, leading to AD (Li et al., 2019). Iron has been reported to accelerate aggregation and pathogenicity of AD-related aberrant proteins, such as β-amyloid, tau, α-synuclein, and TDP (Ashraf and So, 2020). Another study in 2021 found that the loss of Fpn (ferroportin, an iron exporter) in AD mice led to brain atrophy and cognitive impairment, in addition to the morphological and molecular evidence of ferroptosis in hippocampal neurons in Fpn knockout mice (Bao et al., 2021). Restoration of Fpn ameliorated ferroptosis and memory impairment (Bao et al., 2021). Generally, ferroptosis affects AD in terms of iron metabolism, lipid peroxidation, and the glutathione/glutathione peroxidase 4 axis (Chen et al., 2021; Zhang et al., 2021).

Ferroptosis is a unique pattern of cell death that has gained attention in the field of AD research. However, the current studies of AD and ferroptosis lack exploration at the genetic level, although they may help to diagnosis AD and identify therapeutic targets for AD. Therefore, the purpose of the current study was to identify potential biomarkers related to ferroptosis and AD and to provide additional perspective for the research and treatment of AD.

## Materials and methods

### Datasets and pre-processing

Three AD-related microarrays datasets GSE9770 (Readhead et al., 2018), GSE5281 (Liang et al., 2007), GSE28146 (Blalock et al., 2011) were downloaded from GEO (Gene Expression Omnibus) database. Those datasets shared the same platform, GPL570 (HG-U133_Plus_2) (Affymetrix Human Genome U133 Plus 2.0 Array), which showed in the Table 1.

**TABLE 1:** Alzheimer’s disease (AD)-related microarrays datasets in hippocampus.

Dataset	Platform	Total	Alzheimer	Normal
GSE9770	GPL570	34	34	0
GSE5281	GPL570	161	74	87
GSE28146	GPL570	30	22	8

The datasets were processed used R Studio as follows: firstly, after obtaining the raw data of the three datasets, gene expression was normalized and log2-transformed using the LIMMA package (Ritchie et al., 2015) for normalization; then, we removed the no-load and duplicated gene probes to obtain the maximal gene expression probes. GSE9770 and GSE5281 datasets were merged using the Rsva package (Leek et al., 2012), and batch effects were removed. Finally, the LIMMA (Ritchie et al., 2015) package was used to identify DEGs (differentially expression genes) with a log fold change >1 and *p*-value <0.05. The GSE 28146 dataset underwent the same processing flow with the exception of being merged with any other datasets. DEGs were adjusted by the Benjamini–Hochberg method to process the *p*-values.

### The ferroptosis-related genes from FerrDb and hub-genes from Cytoscape by protein–protein interaction

FerrDb^1^ is the world’s first database related to ferroptosis that includes genes and substances (Zhou and Bao, 2020). Ferroptosis-related genes, including driver, suppressor and inducer genes, and we removed the duplicated genes, which finally obtain the total 259 genes. These genes intersect with the DEGs from the union set of GSE9770 and GSE5281 and can be visualized in the Venn diagram. After obtaining the shared genes, STRING^2^ was used to obtain the PPI (protein–protein interactions) with a 0.4 (medium confidence) minimum required interaction score. Cytoscape V3.9.1 software (Shannon et al., 2003) was then used to visualized the network. Moreover, CytoHubba was used to calculate and visualize the top 10 nodes.

### The gene ontology and Kyoto Encyclopedia of Genes and Genomes enrich analysis

The 10 selected genes were used to perform GO (Gene Ontology) analysis to determine BP (biological process), CC (cellular component), and MF (molecular function) term enrichment and perform KEGG (Kyoto Encyclopedia of Genes and Genomes pathway) enrichment. Final significant genes also underwent GO and KEGG enrichment analyses.

### Identifying the significant genes and find the clinical and diagnosing value of biomarkers

The significance of the 10 selected genes were checked again by comparing GSE28146 and the union dataset of GSE9770 and GSE5281 and visualized, respectively. Then, ROC monofactor analysis was performed in these two datasets to find the clinical and diagnostic value of ferroptosis-related biomarkers in AD patients. In the GSE28146 dataset, significant genes were identified not only by expression changes in AD but were also associated with the stage of dementia, which provided phenotype information.

## Results

### Finding and identifying the ferroptosis-related genes

**FIGURE 1:**
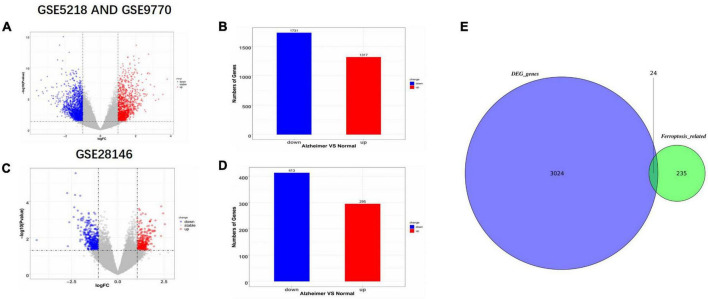
Identification of differentially expression genes (DEGs) and ferroptosis-related genes. **(A,B)** Volcano plot and bar plot of AD and non-AD patients in GSE5218 and GSE9770 after merging and removing any batch effects. **(C,D)** Volcano plot and bar plot of AD and non-AD patients in GSE28146. **(E)** Venn diagram showing the intersection of ferroptosis-related DEGs in the combined set. AD, Alzheimer’s Disease; DEGs, differentially expressed genes.

Three datasets, GSE9770, GSE5281, and GSE28146, were downloaded from the GEO database and processed using the LIMMA package (Ritchie et al., 2015) in R studio. First, the GSE9770 and GSE5281 datasets were combined as a union set to identify ferroptosis-related DEGs, after removing no load or duplicated probes and batch effects. Figures 1A,B shows the up-regulated and down-regulated genes in the union set as volcano plots. The log fold change was set at 1.0 and *p*-value at <0.05. As a result, there were 1,731 down-regulated genes and 1,317 up-regulated genes. GSE28146 was similarly processed, and the results are shown in Figures 1C,D. There were 413 down-regulated genes and 295 up-regulated genes, with a 1.05 log fold change and *p*-value <0.05. The total 235 ferroptosis-related genes obtained from the FerrDb database (see text footnote 1) included driver, suppressor, and inducer genes. In Figure 1E, the Venn diagram shows the union sets and the ferroptosis-related genes. Finally, 24 ferroptosis-related genes were identified as biomarker candidates.

### Protein-protein interaction network and confirming the hub genes

**FIGURE 2:**
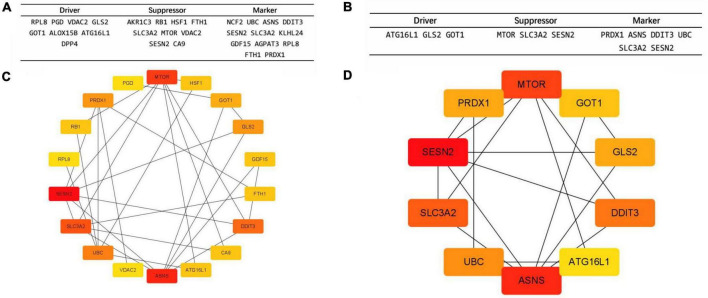
The protein–protein interaction (PPI) network and 10 hub gene interaction network analyzed using Cytoscape software. **(A,C)** The 24 Ferroptosis-related DEGs analyzed using STRINGS and Cytohubba (MCC Ranking method). The network includes 18 nodes and 29 edges. (Disconnected nodes are hidden). **(B,D)** Picking the top 10 rank genes (MCC Ranking method). The network includes 10 nodes and 16 edges. Gene to gene interactions are depicted in the graph as edges between two nodes. The different nodes represent the variation of the degrees from high to low, determined by the degree of interaction.

The names and the categories of the 24 ferroptosis-related biomarker candidate genes are shown in Figure 2A. The STRING database was used to analyze the PPI network of these genes and Cytoscape V3.9.1 was used for visualization of the network (Figure 2C). Cytohubba was applied to calculate the 10 nodes ranked the highest when using the MCC method (Figure 2D). *SESN2, ASNS, MTOR, SCL3A2, DDIT3, UBC, GLS2, PRDX1, GOT1*, and *ATG16L1* were identified as hub genes and are shown with their categories in Figure 2B.

### Gene Ontology and Kyoto Encyclopedia of Genes and Genomes enrichment analysis

**FIGURE 3:**
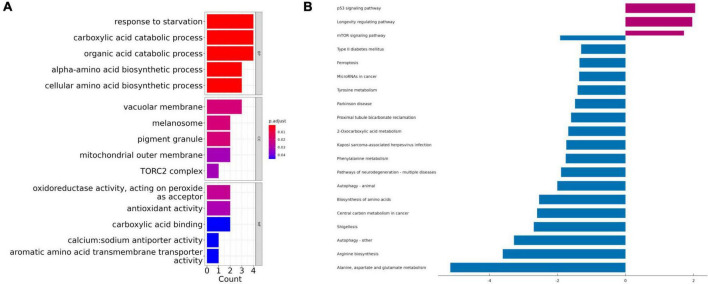
The Gene Ontology (GO) and Kyoto Encyclopedia of Genes and Genomes (KEGG) enrichment analyses using the top 10 ranked ferroptosis-related DEGs. **(A)** Gene Ontology (GO) functional analysis shows enriched items in the top 10 ranked ferroptosis-related DEGs. **(B)** Kyoto Encyclopedia of Genes and Genomes (KEGG) analysis shows the enriched items in the top 10 ranked ferroptosis-related DEGs. BP, biological process; CC, cellular component; MF, molecular function.

Gene Ontology term enrichment was determined for the 10 biomarker candidates and included analysis for enriched BP, MF, and CC (Figure 3A). KEGG enrichment analysis was also performed. The GO-BP results showed that these biomarkers are strongly associated with the “response to starvation”, “carboxylic acid catabolic process”, and “organic acid catabolic process”, compared with other process. In the GO-CC, overlapping genes were mostly enriched in the “vacuolar membrane”, “melanosome”, and “pigment granule” terms. The three most enriched terms in GO-MF were “oxidoreductase activity”, “antioxidant activity”, and “carboxylic acid binding”. Furthermore, KEGG results illustrated that these hub genes were mainly enriched in the “p53 signaling pathway”, “longevity regulating pathway”, “mTOR signaling pathway”, “type 2 diabetes mellitus”, and “ferroptosis” pathways (Figure 3B).

### The expression of overlapped genes and the clinical value in union set

**FIGURE 4:**
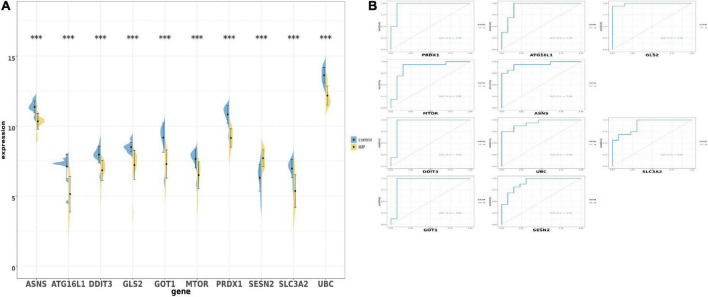
The boxplot and violine plot of the expression levels of the top 10 ranked ferroptosis-related differentially expression genes (DEGs), aimed at finding the significance and the ROC curve to illustrate the diagnostic value of ferroptosis-related DEGs by monofactor analysis. **(A)** Expression of the 10 ferroptosis-related genes compared with the non-AD group within the hippocampus region in AD in the combined with GSE5281 and GSE9770 datasets. **(B)** The AUC in the top 10 ranked ferroptosis-related genes in ROC curve in the set combined with GSE5281 and GSE9770. HIP, hippocampus region in Alzheimer’s disease; ***, *p* < 0.001. AUC, Area under the curve.

After obtaining the 10 ferroptosis-related genes, we were going to verify whether these genes could have same trend of expression. Firstly, we detected the expression of those 10 overlapped genes in union set and performed by boxplot and barplot. The graph showed that all genes is *p* < 0.01. Surprisingly, all genes showed that the expression is down-regulated in hippocampal region in AD patients vs. health person, except the SESN2 which show the opposite trend (Figure 4A). We also used ROC monofactor analysis to perform the diagnostic value of overlapped genes and calculate the AUC area to evaluate the accuracy of these genes’ ability in prediction (Figure 4B). In the plot, we could see that the lowest AUC area is 0.90 and the maximal AUC is close to the one. As the result, the 10 genes are qualified to select as the biomarker in further verification.

### The expression of overlapped genes and the clinical value in GSE28146

**FIGURE 5:**
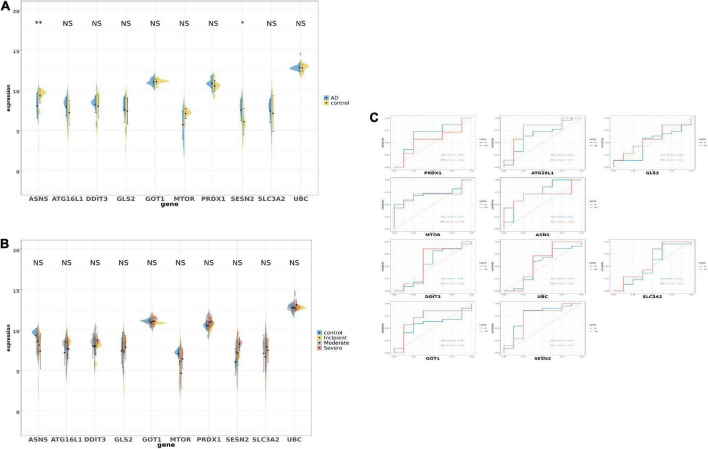
The boxplot and violine plot of the expression in the top 10 ranked ferroptosis-related DEGs, aimed at finding the significance and the receiver operator characteristic (ROC) curve in order to illustrate the diagnostic value of ferroptosis-related DEGs by monofactor analysis. **(A)** The expression of the 10 ferroptosis-related genes in AD compared with the non-AD group within the hippocampus region in GSE28146. **(B)** The expression of the 10 ferroptosis-related genes in AD compared with the non-AD group within the hippocampus region in the different stages of dementia in GSE28146. **(C)** AUC in the top 10 ranked ferroptosis-related genes in the ROC curves in GSE28146. *p < 0.05; **p < 0.01; NS, not significant, *p* ≥ 0.05, non-AD control vs. AD; m2, control vs. incipient vs. moderate vs. severe.

After selecting the 10 genes, the GSE28146 dataset was used to verify results. In Figure 5A, boxplots and bar plots of the 10 eligible genes are shown. Surprisingly, only two genes were significantly changed (*ANSN p* < 0.01, SENS2 *p* < 0.05) in AD patients vs. people without AD. *ASNS* and *SESN2* expression levels in the GSE28146 dataset were similar to that seen in the union set. Phenotypic subgroups were created according to the stage of dementia. There were no gene differences within the subgroups (Figure 5B). ROC monofactor analysis was used to evaluate clinical value for candidate genes. In Figure 5C, the AUC of *ASNS* and *SESN2* was greater than 0.7 in the m1 curve, and *ATG16L1*, *GOT1*, and *SESN2* exceeded 0.7 in the m2 curve. Thus, *ASNS* and *SESN2* were chosen as biomarker candidates with potential diagnostic value in AD.

### The potential mechanism in the selected genes and co-expressed protein network

**FIGURE 6:**
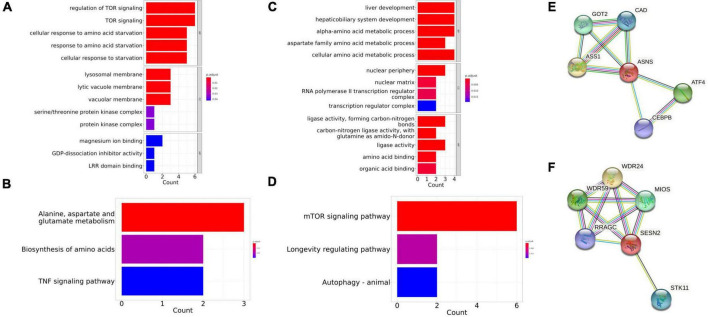
Gene Ontology (GO) and Kyoto Encyclopedia of Genes and Genomes (KEGG) enrichment in the co-expressed protein network of ASNS and SESN2. **(A,B)** The GO and KEGG enrichment of the co-expressed protein network of SESN2. **(C,D)** The GO and KEGG enrichment of the co-expressed protein network of ASNS. **(E)** Co-expressed protein network of ASNS. **(F)** Co-expressed protein network of SESN2.

The STRING database was used to identify the co-expressed protein network of *ASNS* and *SESN2*, and the networks were visualized (Figures 6E,F). Five interactors were identified in these two graphs, respectively. *GOT2*, *CAD*, *ASS1*, *ATF4*, and *CEBPB* are co-expressed with *ASNS*. GO and KEGG analysis results of these six genes are presented in Figures 6C,D. The GO and KEGG enrichment analysis for the co-expressed protein network of *SESN2*, based on the six related genes, is also shown (Figures 6A,B).

## Discussion

Aging consists of many processes, including loss of protein homeostasis, DNA damage, lysosomal dysfunction, epigenetic changes, and immune dysregulation, and it does not mutually and linearly relate to disease (Wyss-Coray, 2016). As normal aging, neurodegeneration and dementia, and cognitive impairment occur along a continuum, it is hard to distinguish the progression from normal aging to disease aging and to find a reliable tool to differentiate these processes, making this area of research a tough and difficult topic (Wyss-Coray, 2016). For the exploration of aging and neurodegenerative diseases, identifying specific cell death patterns and activated signaling pathways in the pathology of disease is crucial (Andreone et al., 2020). Ferroptosis, which is different from other cell death patterns, is a new horizon for scientists to explore novel biomarkers and therapeutic targets (Andreone et al., 2020). A large cohort study showed that the existence of iron has a strong relationship with the mechanisms involved in neurodegeneration diseases, encouraging research into the interactions and associations between AD and ferroptosis (Ayton et al., 2021). Exploring the relationship between ferroptosis and AD may lead to identification of new biomarkers for AD diagnosis and new targets for AD treatment.

More and more scholars are studying the field of ferroptosis and neurodegenerative diseases. An animal study suggested that *SCL40A1*, a ferroptosis-related gene, is associated with cognitive impairment in type I diabetes (Hao et al., 2021). Moreover, another study found that eriodyctiol produces an anti-ferroptosis effect, thereby alleviating cognitive impairment, and the mechanism of these effects may be related to activation of the Nrf2/HO-1 pathway (Li et al., 2022). In the present study, ASNS and SESN2 were significantly related to AD compared with other ferroptosis-related genes. At present, most studies have focused on the role of ASNS in cancer-related studies, including studies on ovarian cancer, oral squamous cell carcinoma, colorectal cancer cell, T-cell acute lymphoblastic leukemia, and skull base chordoma (Lorenzi et al., 2006; Du et al., 2019; Fu et al., 2021; Shen et al., 2021; Akahane et al., 2022). *ASNS* may be a predictive biomarker in those tumors or cancers; however, assays also indicate an association between *ASNS* and neurodegeneration diseases. *SENS2* shows a similar trend to *ASNS* findings. In the study of carcinoma, *SENS2* was identified as a tumor suppressor gene (Weng et al., 2018). More specifically, *SESN2* may be a prognostic biomarker for hepatocellular cancer (Chen et al., 2017). A mouse study using *SESN2/3* knockout mice may allow characterization of its role in aging and metabolic-related disorders. Furthermore, *SESN2* may inhibit the production of ROS and produce antioxidant effects, making it a potential target for the treatment of AD and neurodegenerative diseases, as AD neuronal death is likely caused by oxidative damage (Wang et al., 2019). Surprisingly, a study for AD and mild cognitive impairment patients has suggested that SESN2 plays an important role in the progression of AD (Rai et al., 2016). In summary, the association between ferroptosis and AD may uncover new insights into diagnosis and therapeutic targets for AD. For example, iron chelators, co-drugs and the antioxidant vitamin E, show effects in the treatment of AD (Yuan et al., 2021). However, both biomarkers and therapeutic drugs need to be confirmed by clinical trials.

In conclusion, after processing and validating, ASNS and SESN2 were identified, which pays little attention on the correlation with AD. In our study, ASNS and SESN2 was regarded as the candidate biomarker of diagnosis of AD and even the therapeutic target in the further study. However, we fail to find the gene providing the clinic value of distinguishing the stage of dementia. Because more ferroptosis-related genes have been finding and more hippocampal region samples are needed.

There are several limitations to the current study. First, the datasets chosen and used for analysis—specifically the merged dataset GSE9770–did not provide specific dementia state information. Therefore, there may be limitations regarding AD patient selection and access to DEGs. In addition, because there are few microarrays in the hippocampus of AD patients and the hippocampus of people without AD, only the three datasets used in the present study were available for continuous verification. Finally, the overlapped genes verified in this study need to be supported by future studies and verified by laboratory evidence.

## Data availability statement

The datasets presented in this study can be found in online repositories. The names of the repositories and accession numbers can be found in the article/supplementary material.

## Author contributions

All authors listed have made a substantial, direct, and intellectual contribution to the work, and approved it for publication.

## Footnotes

1
http://www.zhounan.org/ferrdb/current/

2
https://cn.string-db.org/
